# Perforated gangrenous ileo-colic intussusception in a 9 month old Nigerian infant presenting at a private hospital: A case report

**DOI:** 10.1016/j.ijscr.2019.05.007

**Published:** 2019-05-16

**Authors:** Emmanuel Oluchukwu Ani, Lawal Barau Abdullahi, Emmanuel Ajuluchukwu Ugwa

**Affiliations:** a Department of Community Medicine, Bayero University Kano/Zoputa Specialist Hospital, 10 Kabba Street, Nomansland Kano, Nigeria; b Department of Surgery, Aminu Kano Teaching Hospital/Bayero University Kano, Nigeria; c Monitoring, Evaluation, Accountability and Learning Manager, Save the Children International, Abuja, Nigeria

**Keywords:** Perforated gangrenous, Ileo-colic intussusception, Infant, Nigerian

## Abstract

**Introduction:**

Intussusception is the process of invagination of a bowel segment into the adjoining intestinal lumen which may cause bowel obstruction and gangrene. It commonly occurs in infants at a mean age of 9-months with male preponderance. The condition has excellent prognosis if diagnosis is made early and appropriate treatment commenced and mortality rate from intussusception in children can be less than 1%. However, if diagnosis or treatments are delayed it can be fatal in a few days.

**Presentation of case:**

We present a case of Ileo-colic perforated ileum due to delayed diagnosis. It was initially misdiagnosed as gastroenteritis at another hospital. The infant was resuscitated with intravenous fluid and had laparotomy. A signed consent was obtained before the surgery and media consent was signed for publication. A gangrenous terminal ileum was resected and ileo-colic anastomosis was done. Post-operative course was uncomplicated and the patient was discharged after 5days.

**Discussion:**

The index case presented late with complication of gangrene and perforation because it was first managed as a case of gastroenteritis. Gastroenteritis is among the various differential diagnosis of intussusception. Complications have been reported to increase numbers of surgical treatment and sometimes mortality, but rarely occur with good diagnostic acumen.

**Conclusion:**

We conclude that high clinical suspicion, interaction with senior surgeons and regular use of ultrasound in infants with gastrointestinal symptoms will aid diagnosis. Although surgery was performed in the index case, non-surgical reduction is a very efficient treatment modality in uncomplicated cases.

## Highlights

• The current case presented late with complication of gangrene and perforation because it was first managed as a case of gastroenteritis.
• Gastroenteritis is among the various differential diagnosis of intussusception a high index of suspicion is required for timely diagnosis.
• Being available, cheap and free of radiation, using ultrasound more often in infants can aid diagnosis.

## 1 Introduction

Intussusception is the process of invagination of a bowel segment into the adjoining intestinal lumen which may cause bowel obstruction and gangrene. It has been reported among neonates and adults [1,2] but like in the index case and others It commonly occurs in infants at a mean age of 9-months [3,4]. The condition has excellent prognosis if diagnosis is made early and appropriate treatment commenced [5] and mortality rate from intussusception in children can be less than 1% [6]. However, if diagnosis or treatment is delayed it can be fatal in a few days [7]. We report an uncommon case of perforation of a gangrenous intussusception due to delayed diagnosis in a private clinic and underscore the value of interaction with senior surgeons and utilization of ultrasound to solve diagnostic dilemma. The work has been reported in line with the SCARE criteria [8].

## 2 Presentation of case

A 9 month old infant brought into the facility by his parents with history of vomiting and passage of loose mucoid stool for 5 days. There was abdominal distention for 3 days, high-grade fever for 1day and generalized tonic-clonic seizures of 2 h duration prior to presentation. The patient was apparently well until about 5 days prior to presentation when he started vomiting and this was said to be projectile, bilious, non-bloody; about 15 mls; 3–6 bouts per day. At about the same time, patient started passing loose stool which was initially bloody; larger volume and subsequently became mucoid with very little volume and about 3–4 times per day. Three days after the onset of vomiting, parents noticed a gradual increase in the abdominal size, the increase continued till the size patient presented with. A day prior to presentation, patient developed fever which was sudden in onset, continuous, relieved temporary by tepid sponging and administration of analgesics. This was associated with rigor. About two hours prior to presentation, patient started having seizure, which was described as being of the generalized tonic-clonic pattern. He is not a known seizure disorder patient and had no family history of seizure disorder. There was no history of post-ictal sleep or loss of sphincteric control.

For the above complaints, the patient was taken to two health facilities where he was managed as a case of gastroenteritis and they administered antibiotics, analgesics and intravenous infusion that did not relief the symptoms hence his referral for further management and evaluation.

The pregnancy, delivery and neonatal periods were uncomplicated. There was no congenital anomaly or birth defects were noted. He was not exclusively breast fed. He received the routine immunization for his age but has not received rotavirus vaccine. There was no history of surgeries in the past.

**Fig. 1:**
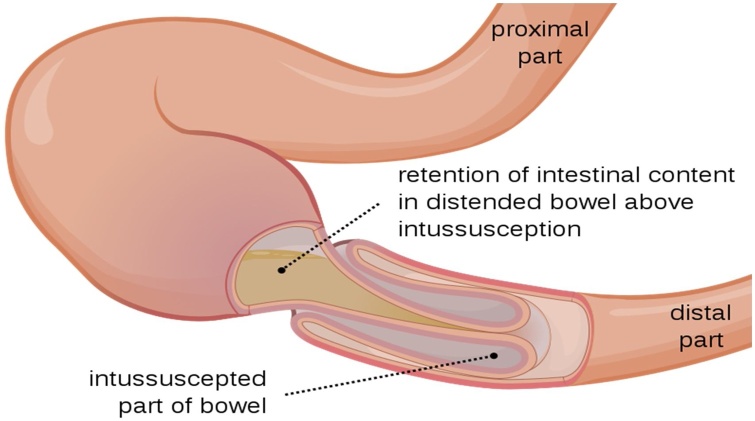
Schematic appearance of intussuseption.

On examination, we saw a male infant who was in respiratory distress (with a respiratory rate of 55 cycles/min), not pale, febrile (38.8 °C), not jaundiced, no pedal oedema, not cyanosed, no significant lymphadenopathy and was severely dehydrated. He had generalized tonic-clonic seizures which resolved on administration of phenobarbitone but there was no focal neurologic deficit. The abdomen was grossly distended, tense and tender with visible dilated abdominal veins, moved minimally with respiration, but there were no palpable organs or masses. The bowel sound was hyperactive. The other systemic examinations were within normal findings (Fig. 1).

The managing doctors interacted and agreed that a diagnosis of generalized peritonitis secondary to gangrenous intussusception was mostly likely judging by the clinical presentations.

Some laboratory investigations and ultrasound was requested.

The full blood count with differential count was normal (packed cell volume – 35%; total cell count – 7.0 × 10^9^/l; neutrophil – 36%; lymphocytes – 62%; eosinophil – 19; basophiles – 19%)

The random blood glucose was 5.3 mmol/, malaria parasite: positive. Blood group: A+, virology were non-reactive, serum electrolyte, urea and creatinine (creatinine – 99.7 umol/l; potassium – 2.0 mmol/l; urea – 3.5 umol/l; sodium – 136 mmol/l; chlorine – 100 mmol/l; bicarbonate – 28 mmol/l) which suggested hypokalemia

**Fig. 2:**
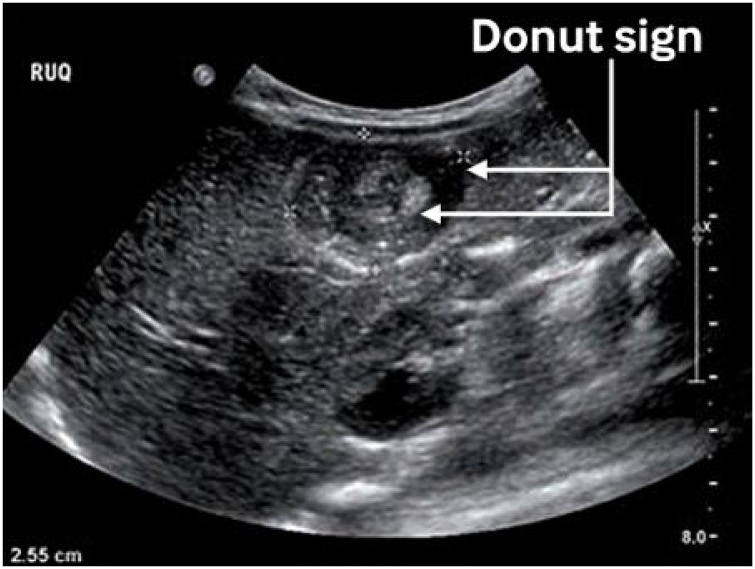
Abdominal ultrasound finding showing characteristic donut appearance.

The abdominal ultrasonography showed a characteristic donut appearance consistent with intussusception and moderate intra-abdominal fluid collection (Fig. 2).

A diagnosis of gangrenous intussusception and hypokalemia was made.

The parents were counseled that surgery will be done and reassured, while their infant was resuscitated with intravenous fluids and had antibiotics. He also had a complete course of antimalarial.

**Fig. 3:**
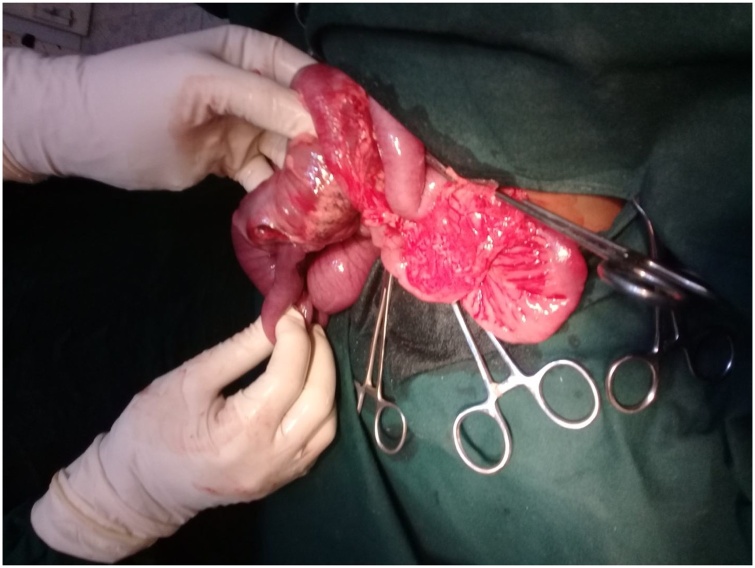
Intussusception being miked out of the intussuscipiens.

**Fig. 4:**
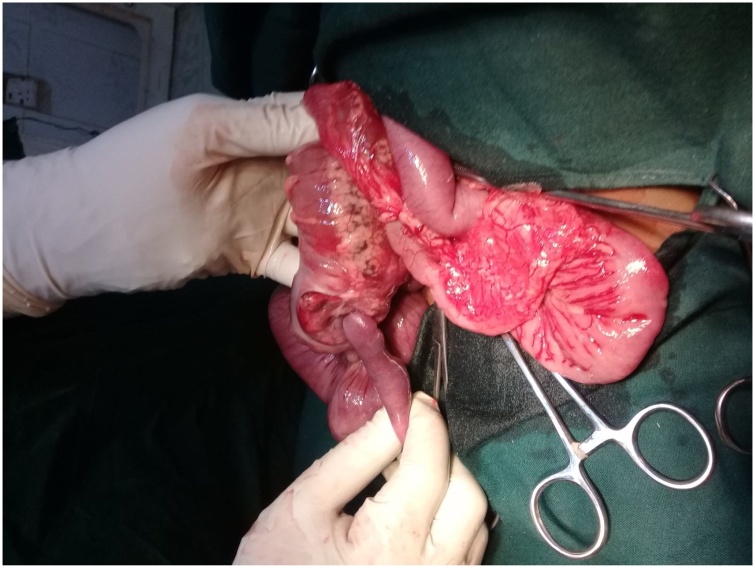
Gangrenous and necrotic bowel segment.

**Fig. 5:**
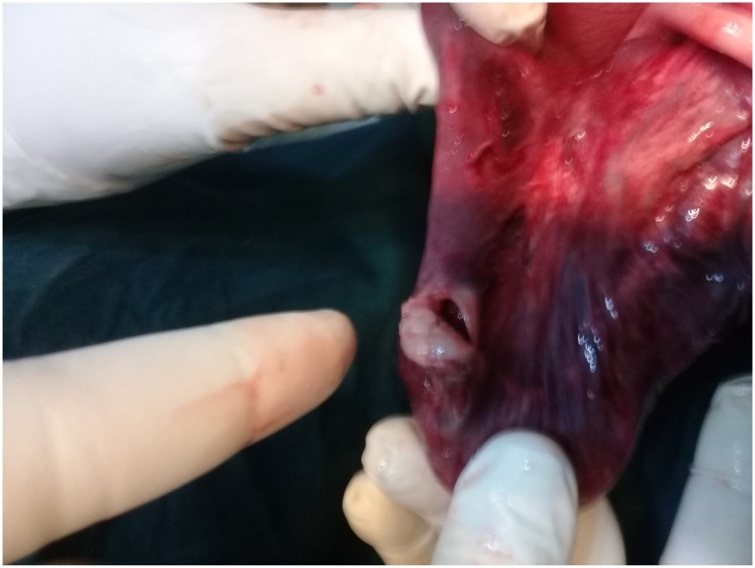
Perforation of the bowel segment from prolonged ischemic necrosis.

A signed consent was obtained and he had an emergency exploratory laparotomy. Open laparotomy and end-to-end ileocolic anastomosis was done. The patient was placed in supine position. Under general anaesthesia plus endotracheal intubation, the abdomen was accessed by a transverse supraumbilical incision.The findings were those of ileo-colic intussusception with gangrenous terminal ileum, caecum and appendix. A perforation was observed at the ileal segment with fecal matter. Resection of these affected segments was done. A handsewn end-to-end anastomosis was effected by placing a stay suture through the antimesenteric border of both ends of the bowel. This helps align the ends and allows the surgeon to keep track of the suture process. Two absorbable sutures were placed next to each other through the mesenteric border of both ends of the bowel. The ends of the two sutures were then tied together. Each suture was then ran toward the antimesenteric border of the bowel, with one suture anastomosing the back wall of the bowel while the other one anastomoses the front wall of the bowel. Care was taken to take full-thickness bites ensuring mucosal inversion along the mesentery as this area was more difficult to visualize than the remaining bowel wall. Inserting the needle on the serosa deeper than the mucosal exit site helped invert the mucosa. Once the two sutures met on the antimesenteric border of the bowel and are tied, the stay suture was removed. The abdomen was closed in layers. The wound was cleaned and dressed. The immediate post-operative condition was stable (Fig. 3, Fig. 4, Fig. 5).

The patient was placed on intravenous, antibiotics, intravenous infusion, analgesics, oxygen therapy by face mask and nil per os until bowel sound returned. Urine output was monitored. The patient maintained a stable post-operative course and was discharged home after 5 days post-surgery.

A parental consent was also obtained and signed for publication.

## 3 Discussion

The current case presented late with complication of gangrene and perforation because it was first managed as a case of gastroenteritis. The differential diagnosis for intussusception includes gastritis, acute appendicitis, hernia, testicular torsion, and volvulus [9,10] but a good clinical acumen through a thorough systemic examination, timely employment of senior surgeons and ultrasound scanning will easily give away the diagnosis and avoid complications [[11], [12], [13], [14], [15]]. The classic clinical triad of intussusception colicky abdominal pain, vomiting and bloody stools is seen in only about 20% of cases [13], a high index of suspicion is required for timely diagnosis.

The findings were those of ileo-colic intussusception with perforated gangrenous ileum. Gangrene of bowel segment in this case is as a result of delayed diagnosis. It has been similarly reported in few studies [[16], [17], [18]]. It can also increase the cases of surgeries, surgical complications and mortality [19,20].

Treatment includes both nonsurgical and surgical procedures; the two methods of nonsurgical reduction are hydrostatic and pneumatic [21]. Nonsurgical reduction can be done safely if there are no contraindications. Absolute contraindications such as peritonitis, perforation, and dehydration lead to nonresponsive shock [22]. Surgical treatment is necessary in cases with contraindications or failed nonsurgical reduction. As in the index case, an emergency exploratory laparotomy with resection and ileo-colic anastomosis was done. Other treatment modalities have been reported with various outcomes [[23], [24], [25], [26], [27]].

The value of ultrasound as in this case cannot be overemphasized. It is available, cheap and free of radiation. The technique is easily learned by non-specialists in resource poor settings. Ultrasound can also be used to guide treatment and monitor responses [28,29]. The authors believe that using ultrasound more often in infants can aid diagnosis.

The patient maintained a stable post-operative course and was discharged home after 5 days post-surgery. Although prognosis is usually better with early presentation and treatment [30,31], the outcome as in the index case is also very good if resuscitation and the following post-operative care is optimum [[32], [33], [34]].

While surgery remain the mainstay of treatment for complicated disease, lack of specialized facilities and trained personnel for nonsurgical reduction have been noted as reason for increase surgical reduction especially for simple cases presenting early [35]. As in the index case, surgical management mainly entails open laparotomy in developing countries where there is limited experience with laparoscopy, but case-series and retrospective studies indicate that exploratory diagnostic laparoscopy may be done in an emergency setting [36,37] and therapeutic laparoscopy may be just as effective as open laparotomy and result in a shorter length of hospitalization [[38], [39], [40]].

## 4 Conclusion

We conclude that high clinical suspicion, interaction with senior surgeons and regular use of ultrasound in infants with gastrointestinal symptoms will aid diagnosis. Although surgery was performed in the index case, non-surgical reduction is a very efficient treatment modality in uncomplicated cases.

## Conflicts of interest

The authors have no conflicts of interest to declare.

## Funding

The authors have received no funding for their research.

## Ethical approval

Ethical approval was obtained from Human Research Ethics Committee, Kano with reference number MOH/Off/797/T.1/1164.

## Consent

Written informed consent was obtained from the guardian for publication of this case report and accompanying images. A copy of the written consent is available for review by the Editor-in-Chief of this journal on request.

## Author contribution

**Study concept or design:** Emmanuel Ani and Emmanuel Ugwa

**Data collection, data analysis or interpretation:** Emmanuel Ani, Lawal Abdullahi, Emmanuel Ugwa.

**Writing the paper**: Emmanuel Ugwa, Emmanuel Ani, Lawal Abdullahi

## Registration of research studies

This study has not been registered.

## Guarantor

Dr Emmanuel Ugwa.

## Provenance and peer review

Not commissioned, externally peer-reviewed
